# *Endozoicomonas* genomes reveal functional adaptation and plasticity in bacterial strains symbiotically associated with diverse marine hosts

**DOI:** 10.1038/srep40579

**Published:** 2017-01-17

**Authors:** Matthew J. Neave, Craig T. Michell, Amy Apprill, Christian R. Voolstra

**Affiliations:** 1 Red Sea Research Center, Division of Biological and Environmental Science and Engineering (BESE), King Abdullah University of Science and Technology (KAUST), Thuwal, Saudi Arabia; 2 Woods Hole Oceanographic Institution, Woods Hole, Massachusetts, USA

## Abstract

*Endozoicomonas* bacteria are globally distributed and often abundantly associated with diverse marine hosts including reef-building corals, yet their function remains unknown. In this study we generated novel *Endozoicomonas* genomes from single cells and metagenomes obtained directly from the corals *Stylophora pistillata, Pocillopora verrucosa, and Acropora humilis*. We then compared these culture-independent genomes to existing genomes of bacterial isolates acquired from a sponge, sea slug, and coral to examine the functional landscape of this enigmatic genus. Sequencing and analysis of single cells and metagenomes resulted in four novel genomes with 60–76% and 81–90% genome completeness, respectively. These data also confirmed that *Endozoicomonas* genomes are large and are not streamlined for an obligate endosymbiotic lifestyle, implying that they have free-living stages. All genomes show an enrichment of genes associated with carbon sugar transport and utilization and protein secretion, potentially indicating that *Endozoicomonas* contribute to the cycling of carbohydrates and the provision of proteins to their respective hosts. Importantly, besides these commonalities, the genomes showed evidence for differential functional specificity and diversification, including genes for the production of amino acids. Given this metabolic diversity of *Endozoicomonas* we propose that different genotypes play disparate roles and have diversified in concert with their hosts.

Many multi-cellular organisms rely on a diverse microbiome to provide important nutritional, protective and developmental functions. These include the transformation of proteins into forms digestible by the host^1^,^2^, synthesis of essential vitamins, minerals or amino acids^3^,^4^, priming of the host immune system^5^,^6^, xenobiotic degradation^7^,^8^, and protection against pathogens^9^,^10^. In higher order vertebrates, such as humans, the microbiome fulfilling these niches is extremely complex and consists of thousands of species and functions, forming an intricate web of interactions^11^. Invertebrates can also form complex symbioses with many microbial partners that provide critical functions for the host. For example, the Hawaiian bobtail squid, *Euprymna scolopes*, uses bioluminescence as a predator-avoidance mechanism through colonization of its light organ by the bacterium, *Vibrio fischeri*^12^. In several beetle species, gut microbes are used to detoxify harmful substances, such as caffeine, or to aid in the digestion of nutrient-poor tissue, thereby contributing to adaptive divergence and niche expansion^1^,^8^. In an example of the protective ability of the microbiome, symbionts of the nematode worm, *Caenorhabditis elegans*, have rapidly evolved mechanisms to protect the host against attacks from invading pathogenic bacteria^10^. The overarching picture that emerges from these and other studies is that animals (and plants) are considered holobionts or metaorganisms that live in close association with a species-specific and diverse microbiome^13^.

Despite these advances in our understanding of the importance of bacterial symbionts to hosts, the function of the overwhelming majority of identified bacteria remains to be determined. One globally distributed group of symbiotic bacteria without a known function are from the genus *Endozoicomonas (Gammaproteobacteria; Oceanospirillales*; see Neave *et al*. for review^14^). These bacteria associate with a wide variety of marine hosts, including corals^15^,^16^,^17^,^18^,^19^, and other cnidarians^20^,^21^, sponges^22^,^23^, gorgonians^24^,^25^,^26^, molluscs^27^,^28^, worms^29^, fish^30^,^31^, and tunicates^32^,^33^. Despite these associations with numerous hosts in oceans worldwide, the functional role of *Endozoicomonas* remains unclear. Dimethylsulfoniopropionate (DMSP) breakdown has been suggested as a potential role^26^,^34^, however, sequenced *Endozoicomonas* genomes lack DSMP metabolic pathways^35^. *Endozoicomonas* may also participate in a nutritional symbiosis, where the bacteria produce extracellular enzymes to degrade complex organic carbon sources that can then be used by the host^25^, as occurs with *Oceanospirillales* bacteria and deep-sea *Osedax* worms^2^. Another possibility is that *Endozoicomonas* interact with the algal symbiont *Symbiodinium*, either in a mutualistic or antagonistic relationship^36^,^37^, although *Endozoicomonas* are also commonly found in organisms without photosymbionts^38^. *Endozoicomonas* may also produce antimicrobial compounds to deter invading pathogenic microbes^39^, which has been seen for other coral-associated bacteria^40^. In contrast to these beneficial scenarios, the only observations of *Endozoicomonas* with marine vertebrates have been with diseased fish in aquaculture facilities. For example, *E. elysicola* formed cysts on the gills of cobia, *Rachycentrum canadum*, causing epitheliocystis and mass mortalities^31^. Moreover, a novel species of *Endozoicomonas* was responsible for epitheliocystis in the sharpsnout bream, *Diplodus puntazzo*^30^. These opposing functions suggest that *Endozoicomonas* have multiple roles in their many hosts, and members from this genus may opportunistically transition through different symbiotic relationships, i.e., mutualistic, commensalistic, and parasitic.

Despite the abundance of *Endozoicomonas* symbionts, only three complete *Endozoicomonas* genomes are publically available, including *E. elysicola, E. montiporae,* and *E. numazuensis*, isolated from a sea slug, coral, and a sponge, respectively^35^,^41^, therefore providing a limited understanding of their functional gene repertoire. The relatively slow pace of *Endozoicomonas* genome sequencing may be attributed to the difficulty in obtaining cultured isolates from host tissue. Here we used culture-independent methods of genome sequencing, including metagenomic binning and single cell genomics, to obtain a further four *Endozoicomonas* genomes from the reef-building corals *Stylophora pistillata, Pocillopora verrucosa*, and *Acropora humilis*. Comparative genomics was subsequently used to collectively interrogate the seven available genomes in order to better understand their shared and distinct functional characteristics. We found that the *Endozoicomonas* genomes were enriched for genes associated with transporter activity, particularly carbon sugar transport, as well as cell secretion and transposase activity, suggesting that *Endozoicomonas* have a potential role in the upcycling of carbohydrates or the supply of proteins to the host. The enrichment in transposase activity may help *Endozoicomonas* to quickly adapt to a new host or take advantage of a new niche. Apart from these commonalities, we also determined the set of taxon-specific genes. Functional enrichment of these species-specific gene sets indicates niche specialization of different *Endozoicomonas* genotypes. This is the first study to comparatively analyse *Endozoicomonas* genomes and provides important functional insight into this enigmatic genus.

## Results

### Genome sequencing and assembly

Metagenomic binning was used to obtain 81.0% of the *Endozoicomonas* genome from *Acropora humilis* and 89.7% of the *Endozoicomonas* genome from *Pocillopora verrucosa*, with low contamination levels for both genomes (Supp. Fig. 1; Supp. Table 1). The genome from *P. verrucosa* in a number of cases contained two copies of expected single copy genes (Supp. Fig. 1; heterogeneity = 2), which was caused by the presence of two *Endozoicomonas* strains that were unable to be separated during the binning process. Difficulties in separating closely related strains is often encountered using metagenomic binning^42^, and for this reason, we restricted our analyses to functional gene content rather than genome size or synteny comparisons to avoid confounding the results.

Using single cell genomics, two distinct strains of *Endozoicomonas* cells were also recovered from the coral *Stylophora pistillata*, designated here as “Type A” and “Type B”. In this case, however, the extraction of single bacterial cells allowed for the two *Endozoicomonas* strains to be sequenced independently. By sequencing and co-assembling 10 identical cells of Type A, 60.2% of the genome was recovered with very little contamination. For Type B, three identical cells were co-assembled, recovering 75.9% of the genome with low contamination (Supp. Fig. 1).

Several limitations to the techniques employed here were experienced, as is commonly encountered, including incomplete genome recoveries, difficulties in separating closely related strains and relatively fragmented genome bins (Supp. Fig. 1; Table 1). For these reasons, our analysis focused on core gene sets or techniques using relative measures rather than absolute (e.g., percent of genes coding for functions, rather than number of genes), thereby minimising the influence of these inherent issues.

### *Endozoicomonas* core genome phylogeny

**Figure 1:**
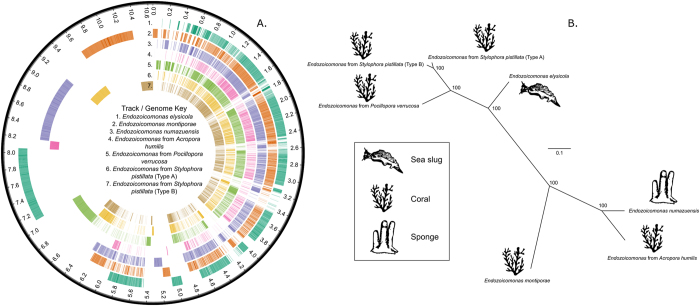
*Endozoicomonas* pan-genome showing (**A**) core and accessory genes, and (**B**) phylogenetic relationship of the *Endozoicomonas* genomes based on core protein sequences. In (**A**), genes shared between genomes are indicated by overlapping segments and the outermost track indicates genome size (million base pairs). In (**B**), the scale bar indicates the mean number of substitutions per site and confidence from 1000 bootstrap replicates are shown on the branches.

A “core” and “accessory” *Endozoicomonas* pan-genome was calculated using all seven genomes (i.e. three that were previously available and four generated in this study) to show regions of genomic similarity and dissimilarity (Fig. 1A). The core set (n = 301) was then used to construct a super-alignment and phylogenetic tree (Fig. 1B). In some cases host phylogeny reflected symbiont phylogeny. For example, the corals *P. verrucosa* and *S. pistillata* belong to the same coral family (*Pocilloporidae*), and their symbionts were closely related (Fig. 1B). Moreover, the *Endozoicomonas* genomes obtained from the same coral species (*S. pistillata* Type A and Type B) were very closely related; in fact, their core amino acid sequences had an average similarity of 97.4%. Interestingly however, the *Endozoicomonas* genomes did not always cluster according to host phylogeny. For example, the *Endozoicomonas* symbiont of the coral *A. humilis* shared a branch with *E. numazuensis*, a sponge symbiont, and was not closely related to the other coral symbionts (Fig. 1B). The remaining *Endozoicomonas* genomes, *E. elysicola*, a sea slug symbiont, and *E. montiporae*, a coral symbiont, did not align closely with any of the other genomes (Fig. 1B).

### Molecule transport and genetic recombination are enriched in *Endozoicomonas* genomes

To determine the functional signatures that characterise the genus *Endozoicomonas*, Gene Ontology (GO) terms were compared between *Endozoicomonas* and other related members of the *Oceanospirillales*, plus more distantly related *Vibrio, Wolbachia* and *Shewanella* bacteria (Tables 2 and 3). We chose these bacterial groups because they contain relatively well-studied symbiotic bacteria and a large number of sequenced genomes. The following comparisons, however, may only be relevant for these particular bacterial groups. Many of the most enriched GO terms were associated with the generic transport of molecules, such as organic substance transport, carbohydrate transport, and single-organism transport. In addition, more than twice the number of genes involved in phosphoenolpyruvate-dependent sugar phosphotransferase (PTS; used for the uptake and phosphorylation of specific extracellular carbohydrates), were detected in *Endozoicomonas* compared to other *Oceanospirillales* bacteria (Table 3). When the genes that comprise the PTS system were examined, 62% of the specific binding components targeted lactose and cellobiose. Another enriched process in *Endozoicomonas* bacteria compared to other *Oceanospirillales* bacteria was dicarboxylic acid transport, which allows for the movement of these molecules within cells and across membranes. Possibly related to this, secretion processes, in particular protein secretion, were significantly enriched in the *Endozoicomonas* genomes compared to other bacteria (Table 3). Another enriched process that may be related to genome adaptability, was transposition (including DNA-mediated) and DNA recombination (Table 3).

### *Endozoicomonas* strains show signs of functional specificity

**Figure 2:**
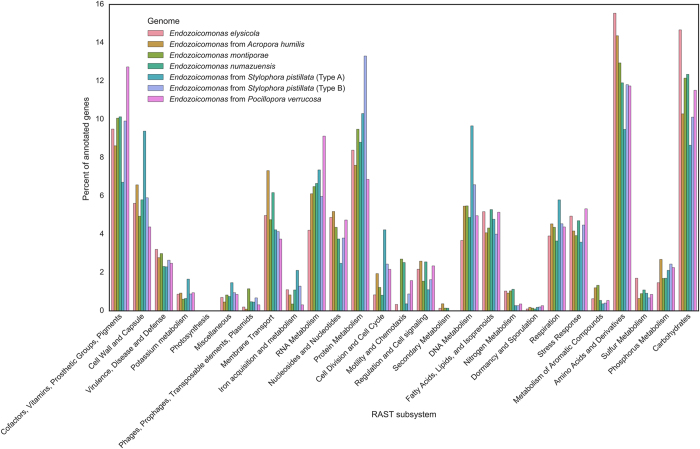
Percentage of *Endozoicomonas* genes annotated into high level functions within the RAST (Rapid Annotation using Subsystem Technology) subsystem classifications.

The *Endozoicomonas* genomes were compared to each other using high-level functions from the RAST subsystem classification, and this corroborated that the *Endozoicomonas* genomes coded for similar high-level functions, although several potential strain-specific functions were detected (Fig. 2; Supp. Table 2). For example, the *Endozoicomonas* from the coral *P. verrucosa* contained more genes for cofactors, vitamins, prosthetic groups, pigments and RNA metabolism, compared to the others. Interestingly, *Endozoicomonas* Type B from the coral *S. pistillata* coded for ~50% more cofactors, vitamins, prosthetic groups, pigments than the very closely related Type A from the same coral (Fig. 2). Within this functional group, 64% of the genes were for riboflavin and folate biosynthesis. In addition, Type A had more genes for DNA metabolism, while on the other hand, the Type B strain had more genes for protein metabolism (Fig. 2; Supp. Table 2). All of the *Endozoicomonas* genomes devoted much of their functional repertoire to carbohydrate metabolism (~10%), however, *E. elysicola*, a sea slug symbiont, had a particularly high percentage (~15%; Fig. 2).

**Figure 3:**
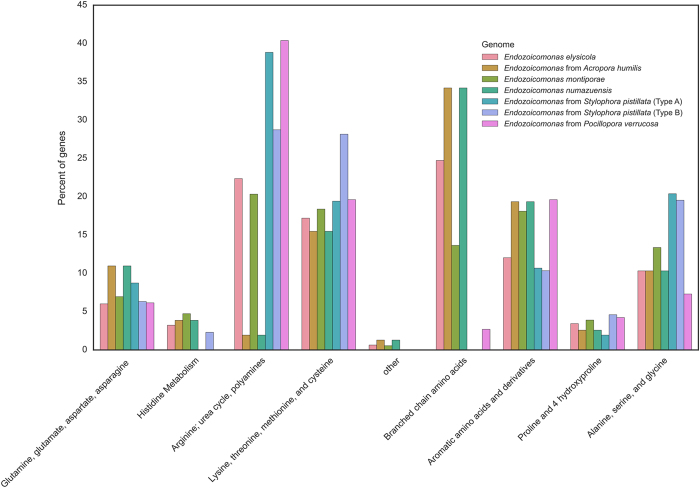
Percentage of *Endozoicomonas* genes in the RAST (Rapid Annotation using Subsystem Technology) amino acids and derivatives classification.

Another category containing a large number of genes was amino acids and derivatives (Figs 2 and 3). This category was examined in more detail due to the interesting possibility that the symbionts produce essential amino acids that cannot be synthesized by the host^4^. Strain variability was seen in the genes encoding arginine, the urea cycle, and polyamines (Fig. 3; Supp. Table 3). In particular, *E. numazuensis* and *Endozoicomonas* from *A. humilis* had very few genes in this category, however, all other genomes were well represented. Moreover, there were further functional divisions within this group. A number of the genomes distributed functions between arginine biosynthesis (*E. elysicola* (33%), *E. montiporae* (44%), *Endozoicomonas* from *P. verrucosa* (44%)) and degradation (*E. elysicola* (46%), *E. montiporae* (48%), *Endozoicomonas* from *P. verrucosa* (45%)). In contrast, the two genomes from *S. pistillata*, Types A and B, did not code any genes for arginine biosynthesis, instead encoding more than 80% of the genes for arginine degradation. Similarly, Types A and B from *S. pistillata* did not encode any genes for branched chain amino acids (Fig. 3; Supp. Table 3), while the other genomes in this category coded for isoleucine, leucine, and valine biosynthesis and degradation. Another interesting amino acid category was alanine, serine, and glycine. In this case, Types A and B from *S. pistillata* coded almost 50% more alanine and serine biosynthesis genes than the other genomes (Fig. 3).

## Discussion

This study compared the genomes of *Endozoicomonas* associated with corals, a sponge and a sea slug obtained from isolates and cultivation-independent metagenomics and single cell sorting. The sequencing and availability of these *Endozoicomonas* genomes from a diverse range of hosts, environments, and ecologies provides a solid foundation for understanding the functional diversity of *Endozoicomonas*, and our analysis provides new insight about their genomic similarities and functional characteristics.

By comparing the phylogenetic relationships of the genomes, patterns of co-diversification between host and symbiont emerged, which has been found for other *Endozoicomonas* symbionts. For example, La Rivière *et al*. found that *Endozoicomonas*-like symbionts in gorgonians had similar phylogenetic relationships to their hosts^43^, suggesting the co-divergence of host and symbiont. Here, the related corals *Stylophora pistillata* and *Pocillopora verrucosa* had symbionts that were also related, potentially indicating co-diversification between host and symbionts. However, symbionts from the other two coral species, *Acropora humilis* and *Montipora aequituberculata*, were not closely related, suggesting that co-diversification if occurring is more complicated and may depend on other factors. For example, Neave *et al*. found that the brooding coral *S. pistillata* contained *Endozoicomonas* genotypes specific to well-defined geographic areas, while the spawning coral *P. verrucosa* shared *Endozoicomonas* genotypes across large geographic scales^44^. Accordingly, differences in the mode of symbiont transmission (i.e. horizontal or vertical) may determine if the symbiont will co-evolve with the host, and account for some of the differences observed here.

The *Endozoicomonas* genomes were enriched for genes involved in the transport of molecules, and genes for the secretion of proteins, when compared to other *Oceanospirillales* bacteria and more distantly related bacterial groups including some symbionts. This enrichment in transport and secretion may relate to the transfer of organic molecules between the symbiont and host, or alternatively, between individuals of *Endozoicomonas* within the cyst-like structures that they typically form^30^,^44^. Of particular interest, dicarboxylic acid transporters were enriched in the *Endozoicomonas* genomes, which has been seen in other symbioses, such as the well-known legume-*Rhizobium* symbiosis^45^. In this case, the plant exchanges carbon photosynthates in the form of dicarboxylic acid for fixed nitrogen in the form of ammonia, which is produced by the symbiotic bacteria^46^. In fact, dicarboxylic acid is the primary carbon source for these symbionts^46^. A similar symbiosis may be at work here between *Endozoicomonas* bacteria and the photosynthate-producing *Symbiodinium* algae. Although none of the *Endozoicomonas* genomes have the genes for fixing nitrogen directly, *E. elysicola, E. numazuensis,* and *E. montiporae*, all have several forms of nitrate reductases, allowing the conversion of nitrate to nitrite and the conversion of nitrite to ammonia, which could then be secreted. Indeed, nitrogen cycling is discussed as one of the key regulatory processes in coral holobiont functioning^47^. Alternatively, the ammonia may be further transformed by the bacteria into useful amino acids. In fact, all of the *Endozoicomonas* genomes contained pathways for the assimilation of ammonia through the synthesis of glutamine and glutamate. Interestingly, in symbioses between pea aphids and *Buchnera* bacteria, glutamine and glutamate are the only precursors required for the synthesis of all other essential amino acids by the *Buchnera* symbionts^4^,^48^. The *Endozoicomonas* genomes contained complete pathways for the synthesis of a variety of amino acids, including alanine, aspartate, cysteine, glycine, homocysteine, homoserine, leucine, lysine, methionine, serine, and threonine. The genomes differed, however, in their capacity to produce these amino acids, which may indicate strain-specific functions. Although the production of essential amino acids may be a role for *Endozoicomonas* symbionts, more research into each specific symbiotic system is required. First steps may include the sequencing of the host genome to determine if essential amino acid biosynthesis pathways are absent.

The *Endozoicomonas* genomes were also enriched for genes involved in the phosphoenolpyruvate-dependent sugar phosphotransferase (PTS) system. This system detects the nutritional requirements of the cell and regulates the phosphorylation and uptake of sugars accordingly^49^,^50^. Interestingly, the PTS system in *Endozoicomonas* mostly encoded for lactose and cellobiose specific subunits. Cellobiose is a basic sugar component of cellulose, which is an important constituent of plant cells, including algal cells^51^. This raises the interesting possibility that *Endozoicomonas*, which may live in symbiotic partnerships with *Symbiodinium* algae, consume degrading algal cells. This process may be beneficial to the host by removing unwanted algal components after cell death. Alternatively, *Endozoicomonas* may live parasitically on algal cells. Indeed, a previous microscopy study detected some *Endozoicomonas* cells in close proximity to *Symbiodinium* cells within a coral host^44^. The PTS system may also be involved in chemotaxis^52^ or the detection of quorum-sensing molecules^53^. As previously discussed, *Endozoicomonas* frequently form cyst-like clusters in their host^30^,^44^ and quorum sensing could provide an important communication channel between individuals. Chemotaxis for the mobile *Endozoicomonas* cells is also likely to be an important process, particularly for finding optimal niche microhabitats within their many hosts.

Another enriched process in the *Endozoicomonas* genomes was transposition (mostly DNA-mediated) and DNA recombination, which may help the species to rapidly adapt to a new host or to opportunistically transition between symbiotic lifestyles (mutualistic, commensalistic, or parasitic). A recently conducted analysis of an *Endozoicomonas* genome that is parasitic on the sharpsnout bream, *Diplodus puntazzo*, also found a high proportion of transposases, which was suggested as a mechanism for adapting to a new niche or host^30^. Importantly, expansion of transposases in the genome, particularly insertion sequences, is thought to be an early step in the transition of a free-living bacterium to a host-adapted lifestyle^54^. For example, the arthropod and nematode endosymbiont, *Wolbachia*, has a significantly reduced genome size with a high proportion of non-functional insertion elements^55^. Almost a quarter (23%) of genes in the obligate intracellular symbiont, *Amoebophilus asiaticus,* code for transposase genes, indicating genome degradation and adaption to its new host^56^. Transposases may also help symbionts by allowing the rapid evolution of mechanisms to avoid host immune responses^57^. Although the *Endozoicomonas* genomes are enriched for transposase elements, the genomes are also relatively large (about 2.8 Mbs and up to 6.3 Mbs; Table 1), suggesting that they are not undergoing streamlining. It’s possible that *Endozoicomonas* strains have a free-living stage, perhaps when moving between hosts, which requires the maintenance of a complete gene repertoire. Different *Endozoicomonas* strains are also likely to have different lifestyles, which could also influence genome structure and restructuring.

In several instances the *Endozoicomonas* species showed signs of functional specificity. For example, the species often differed in their ability to produce certain amino acids, which may relate to what can be consumed from the host, or which amino acids are required by the host. A particularly interesting example of functional specificity was seen in the two *Endozoicomonas* genotypes isolated from the same coral (*Stylophora pistillata*, Types A and B). These two genotypes were very closely related based on their core genome similarity (Fig. 1B), suggesting a recent speciation event. In fact, studies using traditional 3% OTU clustering of the SSU rRNA gene would be unlikely to differentiate these two strains. Nevertheless, the Type A genotype had more genes for DNA metabolism, while Type B had more genes for protein metabolism, possibly indicating niche partitioning within the coral holobiont. Moreover, Type B was enriched for the production of riboflavin and folic acid, two important B vitamins. This production of B vitamins has been seen in other relationships between corals and bacteria and may be an important process for healthy coral functioning^58^,^59^. These functional variations could indicate that the genotypes occupy two different niches within the coral, or alternatively, one genotype may be replacing the other due to the natural selection of beneficial functions. Multiple genotypes of *Endozoicomonas* are often detected within individual hosts, particularly in corals^44^. This seemingly frequent divergence of *Endozoicomonas* genotypes may be facilitated by the high proportion of transposases in the genomes, as discussed above.

The *Endozoicomonas* genomes were obtained using metagenomic binning and single cell genomics techniques due to difficulties in obtaining cultured isolates, and several advantages and shortcomings associated with the techniques were experienced. Metagenomic binning is cost effective as there are few laboratory-processing steps, which may allow more genomes to be obtained. On the other hand, the *in silico* binning process is only becoming established, and still requires time investment and bioinformatics training. Moreover, the binning process is complicated by the presence of closely related genotypes or abundant DNA from other organisms, such as the coral and *Symbiodinium* here, although this may be overcome with the development of new bioinformatics pipelines^60^,^61^,^62^. In this regard, a major advantage of single cell genomics is the ability to confidently isolate and sequence the genome of interest, including genomes from closely related strains. Conversely, single cell genomics can be expensive due to the specialized procedures, and isolated single cells require amplification of their DNA before sequencing (typically using multiple displacement amplification (MDA)), which can lead to amplification bias and problems with genome assembly. We experienced several of these issues, including genome incompleteness, heterogeneity, and uneven genome amplification (due to MDA) that may have non-randomly biased our genome comparison results. Thus, important genes or functions may have been missed in the incomplete *Endozoicomonas* genomes. Nevertheless, we believe that many of these issues were mitigated by the analysis of relative gene set abundances and by comparisons between all seven *Endozoicomonas* genomes with other bacterial genome sequences. Although the techniques used here are valuable for obtaining genomic information, they do not explore the complex dynamics of *Endozoicomonas* bacteria *in situ*. Future studies may use techniques such as single cell RNA-Seq^63^ or secondary ion mass spectrometry (SIMS)^64^ to refine our understanding of *Endozoicomonas* symbiotic relationships and their functional role within the microbiome (see Neave *et al*. for further discussion^14^).

## Conclusions

*Endozoicomonas* bacteria frequently associate with a diverse variety of marine hosts in oceans worldwide. Despite this ubiquity, the specific functional role of *Endozoicomonas* symbionts is unknown. Here we used metagenomic binning and single cell genomics to increase the number of available *Endozoicomonas* genomes. Comparative analysis revealed that *Endozoicomonas* genomes are enriched for transport and secretion processes, which may be related to the transfer of carbohydrates, amino acids, and proteins between the symbiont and host. In addition, many of the enriched processes imply the transfer of molecules between other members of the holobiont. Moreover, the *Endozoicomonas* genomes encoded a large number of transposase genes that may be used to rapidly adapt to a new host or niche. Importantly, *Endozoicomonas* species show signs of functional specificity, in particular with regard to the production of amino acids which may provide insight into specific host requirements. The large functional diversity and plasticity of *Endozoicomonas* genomes suggests diverse functional roles.

## Methods

### Culture isolate sequencing

The genomes of *Endozoicomonas elysicola* from the sea slug *Elysia ornata*^65^, *Endozoicomonas montiporae* from the coral *Montipora aequituberculata*^66^, and *Endozoicomonas numazuensis* from the sponge cf. *Haliclona* spp.^23^ were obtained from a previous publication^35^.

### Coral sampling

Due to unsuccessful attempts to culture *Endozoicomonas* from corals, we used metagenomic binning and single cell genomics to obtain *Endozoicomonas* genomes in a culture-independent manner. These techniques are facilitated by high abundance of the target bacterium; therefore, we used the corals *Stylophora pistillata, Pocillopora verrucosa*, and *Acropora humilis*, which harbor high concentrations of *Endozoicomonas* symbionts in the Red Sea^19^. Samples of each coral were collected in triplicate from Al Fahal Reef, which is located on the Saudi Arabian coast (22°15.100 N, 38°57.386 E). The corals were sampled using SCUBA at depths between 2 and 10 m by removing ~5 cm^2^ fragments with a hammer and chisel. Fragments were placed into Whirl-Pak bags (Nasco, Salida, CA, USA) underwater, brought to the surface, placed on ice and taken to the laboratory, where they were divided into samples for metagenomics (frozen to −80 °C) and single-cell sorting (processed immediately).

### Metagenomic sequencing and binning

The differential coverage binning procedure outlined by Albertsen *et al*. was used with minor modifications to isolate *Endozoicomonas* genomes from other organisms *in silico*^42^. This procedure requires a minimum of 2 metagenomes, in which the target species has different abundances to generate differential coverage profiles. This differential was achieved by sequencing an unmodified metagenome and a size-fractionated metagenome each from *S. pistillata, P. verrucosa*, and *A. humilis*. Tissue was first removed from the coral skeletons by airbrushing with cold 1× PBSE (1× phosphate buffered saline, 10 mM tri-sodium EDTA). A portion of these cells were used directly for DNA extraction to obtain the unmodified metagenome. The fractionated metagenome samples were created by vortexing the airbrushed cells for 1 min, then passing the homogenate through a 5 μm filter, and centrifuging for 15 min at 500 g^67^. The supernatant was collected and centrifuged for a further 20 min at 8,800 g to pellet the remaining cells, which were then resuspended in 200 μl of PBSE. The resuspension was divided into 100 μl aliquots and layered separately over 300 μl of a 26%, 22% and 15% discontinuous Nicodenz gradient (Sigma-Aldrich, St. Louis, MO, USA), before centrifugation at 21,000 g for 60 min. The top 300 μl of the suspension was expected to contain a high percentage of bacterial cells and was used for DNA extraction. Several gradients from the same colonies were required to generate sufficient DNA for sequencing. DNA was extracted from both the fractionated and unmodified samples using the DNeasy Mini Plant Kit (Qiagen Inc., Valencia, CA, USA) according to the manufacturer’s instructions. The proportion of DNA belonging to coral, *Symbiodinium*, and bacteria was tested using a multiplex PCR to ensure adequate recovery of bacterial DNA. The PCR was compiled using the Qiagen Mulitplex PCR kit (Valencia, CA, USA) as per the manufacturer’s instructions, with primers targeting bacterial small subunit (SSU) ribosomal RNA (rRNA) genes (27F/1492R)^68^, the SSU rRNA of *Symbiodinium*, algae (ss3Z/ss5)^69^, and coral mitochondria (LP16S F/R)^70^. Products were screened for size on a 1% agarose gel with a 1 kb ladder (Sigma-Aldrich, St. Louis, MO, USA), and samples with minimal *Symbiodinium* and coral contamination were used for sequencing.

Unmodified and fractionated metagenomes from the corals were sequenced using 1 lane of a 2 * 100 bp, paired-end, Illumina HiSeq run (Illumina, San Diego, CA, USA) (Supp. Table 1). Raw reads were trimmed when the quality per base dropped below 20, and Illumina adapters and reads less than 75 bps were removed using Trimmomatic v.0.33^71^. As per the Albertsen *et al*. binning procedure^42^, the unmodified and fractionated metagenomes were combined and assembled together using IDBA-UD v.1.1.1^72^ with read correction enabled. To generate coverage profiles, reads from the unmodified and fractionated metagenomes were mapped separately to the combined assembly using Bowtie v.2.2.4^73^. Tetranucleotide frequency and GC content of the assembled contigs were calculated with scripts provided by Albertsen and colleagues^42^. Essential single copy genes were detected with Prodigal v.2.6.2^74^, HMMER v.3.0^75^, and MEGAN4^76^. Using these statistics, contigs originating from *Endozoicomonas* genomes were separated from other organisms in R (see Supp. Fig. 2 for example of the binning procedure and Supp. Table 1 for assembled read numbers). Often these metrics were not enough to separate the numerous coral contigs from the bacteria bins, and to increase the discriminatory power we calculated the coding region frequency per contig (expected to be high for prokaryotes, low for eukaryotes) using the earlier results from Prodigal v.2.6.2^74^. Putative *Endozoicomonas* contigs were re-assembled by mapping raw reads to the contigs in Bowtie v.2.2.4^73^, extracting any missing read pairs from the matches and assembling again with IDBA-UD v.1.1.1^72^. A final contamination check was conducted using BLAST against NCBI’s GenBank, and contigs with identities to eukaryotes were removed. Genome completeness and contamination was determined using checkM^77^ and the genome assemblies were annotated using the RAST pipeline^78^. While this procedure yielded adequate *Endozoicomonas* genomes from *A. humilis* and *P. verrucosa*, it was unsuccessful in retrieving *Endozoicomonas* genomes with sufficient completeness from *S. pistillata*. For this reason, we decided to pursue single cell genomics for obtaining *Endozoicomonas* genomes from *S. pistillata* (see below).

### Single cell genomics

Samples from the coral *Stylophora pistillata* were used for a single cell genomics procedure. Immediately after collection, tissue was airbrushed from the coral skeleton using cold PBSE. The coral slurry was divided into 1 mL aliquots, combined with 100 μl of glyTE (10 × Tris EDTA, 50% glycerol), mixed gently for 5 min at ambient temperature, and frozen in liquid nitrogen to −80 °C. Samples were then shipped on dry-ice to the Bigelow Single Cell Genomics Center (SCGC) in Boothbay, ME, USA, and sequenced as described by Stepanauskas and Sieracki^79^. Briefly, the homogenate was sorted using fluorescence-activated cell sorting (FACS) with the sort gate based on side scatter and SYTO-9 fluorescence, and a region was selected based on bacteria-sized particles that formed a relatively homogenous cluster (Supp. Fig. 3). It should be noted that the homogenate was relatively challenging to sort due to the high abundance of other fluorescent particles, which presumably included mitochondria, host cell debris, and other attached bacteria. The selected bacterial cells were then lysed, subjected to multiple displacement amplification (MDA), and screened using amplification of nearly full length bacterial and archaeal SSU rRNA genes followed by direct Sanger sequencing^79^. Of the 384 cells screened, 66 were identified as *Endozoicomonas*, 1 belonged to the Rhodobacteraceae, and the remaining cells did not produce high-quality sequences and therefore could not be identified. Interestingly, 2 distinct strains of *Endozoicomonas* were detected by SSU rRNA sequence similarity (Type A and Type B), and both were selected for whole genome sequencing. For Type A, 10 cells with identical SSU rRNA gene sequences were selected, and for Type B, 3 identical cells were selected. DNA from these cells was sequenced using 1 line of a 2 * 100 bp, paired-end, Illumina HiSeq run and raw reads were trimmed as above using Trimmomatic v.0.33 (Supp. Table 1)^71^. Cleaned reads from each cell type were combined and assembled using SPAdes v.3.5.0^80^ with the single cell flag. Genome assemblies were checked for contamination using the IMG single cell pipeline^81^, which included BLAST similarity checks and identification of outlying contigs based on tetranucleotide frequencies. As previously, genome completeness and contamination was determined using checkM^77^ and the assemblies were annotated using RAST^78^.

### Core genome analysis

The “core” *Endozoicomonas* genome (i.e., genes present in all genomes) was determined by clustering high quality proteins (greater than 10 amino acids in length and less than 20% stop codons) using orthoMCL^82^. The core gene set was extracted from the orthoMCL results using custom scripts in Python v.2.7.5. Detected core protein sequences (n = 301) were then aligned using MUSCLE v.3.8.31^83^ and well-aligned regions were extracted and concatenated into a super alignment with Gblocks v.0.91^84^. An unrooted phylogenetic tree was drawn from the super alignment using RAxML v.8.2.4^85^ with the automatically detected best GAMMA model of rate heterogeneity. An *Endozoicomonas* pan-genome, showing both core and accessory genes (only present in some genomes), was drawn using Circos v.0.69^86^.

### *Endozoicomonas* enrichment analysis

A gene ontology enrichment analysis^87^ was conducted to investigate high-level functions that characterise the genus *Endozoicomonas*. Functional enrichment in the *Endozoicomonas* genomes was tested by comparison to 19 fully sequenced genomes available in GenBank^88^, some of which are close relatives to the *Endozoicomonas*, e.g. *Hahella chejuensis*, and some which are more distantly related, e.g. *Vibrio* species (see Table 2 and Results). All genomes were downloaded and annotated with gene ontology (GO) information using InterProScan v.5.6^89^ and enrichment analysis of the GO terms was conducted using Fisher’s exact tests in the R package topGO v.2.22.0^90^.

## Declarations

### Ethics approval and consent to participate

Experimental research detailed in this study complies with institutional guidelines following KAUST Institutional Biosafety and BioEthics Committee (IBEC).

## Additional Information

**How to cite this article:** Neave, M. J. *et al. Endozoicomonas* genomes reveal functional adaptation and plasticity in bacterial strains symbiotically associated with diverse marine hosts. *Sci. Rep.* **7**, 40579; doi: 10.1038/srep40579 (2017).

**Publisher's note:** Springer Nature remains neutral with regard to jurisdictional claims in published maps and institutional affiliations.

## Supplementary Material

Supplementary Information

## Figures and Tables

**Table 1:** Assembly quality and RAST (Rapid Annotation using Subsystem Technology) annotation results for the *Endozoicomonas* genomes.

Genome	RAST ID	Assembly size (bp)	Contigs	Scaffolds	Scaffold N50 (bp)	Max scaffold size (bp)	CDS	RNAs	GC%
*Endozoicomonas elysicola*^1^	1121862.6	5,569,560	21	2	5,569,560	5,569560	5,021	104	46.8
*Endozoicomonas montiporae*^1^	1027273.4	5,602,297	83	20	1,015,541	1,412,099	5,350	114	48.5
*Endozoicomonas numazuensis*^1^	1137799.4	6,342,227	131	31	917,146	1,695,894	5,995	95	47.1
*Endozoicomonas* from *Stylophora pistillata* (Type A)	6666666.127878	3,624,544	1,553	1,548	10,138	63,630	3,463	55	49.6
*Endozoicomonas* from *Stylophora pistillata* (Type B)	6666666.127879	3,413,810	1,135	1,132	18,779	107,991	3,383	54	50.6
*Endozoicomonas* from *Acropora humilis*	305899.13	2,304,083	1,698	1,698	1,686	8,572	2,142	19	49.4
*Endozoicomonas* (2 genotypes) from *Pocillopora verrucosa*	305899.6	5,277,023	3,342	3,342	2,052	9,019	4,420	42	53.9

^1^*E. elysicola, E. montiporae*, and *E. numazuensis* from Neave *et al*.^35^.

**Table 2:** Genomes used for comparative Gene Ontology (GO) analysis.

Genome	GenBank ID#	Genome size (bps)	Habitat
*Oceanospirillales*			
*Hahella chejuensis* KCTC 2396	PRJNA16064	7,215,267	Sediment
*Hahella ganghwensis* DSM 17046	PRJNA182405	6,564,965	Sediment
*Halomonas halodenitrificans* DSM 735	PRJNA221029	3,464,094	Brine
*Marinomonas ushuaiensis* DSM 15871	PRJNA235145	3,342,098	Seawater
*Oceanobacter kriegii* DSM 6294	PRJNA185608	4,505,834	Seawater
*Oceanospirillum maris* DSM 6286	PRJNA185609	3,709,807	Seawater
*Osedax* symbiont RS1	PRJNA191058	4,505,254	Deep sea *Osedax* worms
*Thalassolituus oleivorans* MIL-1	PRJEB1425	3,920,328	Sediment
*Zooshikella ganghwensis* DSM 15267	PRJNA182446	5,798,664	Sediment
SAR86A	PRJNA76773	1,250,389	Seawater
SAR86B	PRJNA76775	1,749,017	Seawater
SAR86E	PRJNA170317	1,396,800	Seawater
*Other*			
*Wolbachia* sp.	PRJNA272	1,267,782	Fruit fly *Drosophila melanogaster*
*Wolbachia* sp.	PRJNA176303	1,295,804	Fruit fly *Drosophila simulans*
*Shewanella colwelliana* ATCC 39565	PRJNA204100	4,575,622	Sediment
*Shewanella frigidimarina* NCIMB 400	PRJNA13391	4,845,257	Seawater
*Shewanella putrefaciens* 200	PRJNA13392	4,840,251	Seawater
*Vibrio fischeri* ES114	PRJNA12986	4,273,718	Seawater, *Euprymna scolopes* symbiont
*Vibrio fischeri* MJ11	PRJNA19393	4,503,336	Seawater, *Euprymna scolopes* symbiont

**Table 3:** Enriched gene ontology (GO) terms in the biological process category for the *Endozoicomonas* genomes.

Term	Function	Annotated	*Endozoicomonas*	Expected	Fisher’s p-value
*Endozoicomonas vs. all genomes in Table 2*					
GO:0006259	DNA metabolic process	2124	803	607.32	1.5e-21
GO:0006313	transposition, DNA-mediated	450	216	128.67	1.6e-18
GO:0032196	transposition	450	216	128.67	1.6e-18
GO:0006310	DNA recombination	830	342	237.32	2.2e-15
GO:0046903	secretion	424	194	121.24	3.4e-14
GO:0009306	protein secretion	417	190	119.23	1.0e-13
GO:0032940	secretion by cell	417	190	119.23	1.0e-13
GO:0008643	carbohydrate transport	262	130	74.91	4.9e-13
GO:0006024	glycosaminoglycan biosynthetic process	21	21	6	3.8e-12
GO:0033036	macromolecule localization	808	320	231.03	6.4e-12
*Endozoicomonas vs. other Oceanospirillales genomes in Table 2*					
GO:0071702	organic substance transport	1220	648	441.67	<1e-30
GO:0006259	DNA metabolic process	1687	803	610.74	2.1e-23
GO:0008643	carbohydrate transport	183	130	66.25	7.8e-22
GO:0006310	DNA recombination	629	342	227.72	4.5e-21
GO:0006313	transposition, DNA-mediated	357	216	129.24	5.4e-21
GO:0009401	phosphoenolpyruvate-dependent sugar phosphotransferase	107	86	38.74	8.5e-21
GO:0044765	single-organism transport	2661	1180	963.36	4.1e-20
GO:0098656	anion transmembrane transport	78	64	28.24	1.1e-16
GO:0006835	dicarboxylic acid transport	65	55	23.53	1.1e-15
GO:1903825	organic acid transmembrane transport	70	56	25.34	7.5e-14
