# Supplementary material for: Endozoicomonas genomes reveal functional adaptation and plasticity in bacterial strains symbiotically associated with diverse marine hosts
Source: Sci Rep. 2017 Jan 17;7:40579. doi: 10.1038/srep40579 (PMC5240137; doi:10.1038/srep40579)
Supplement: Supplementary Information [file srep40579-s1.pdf]

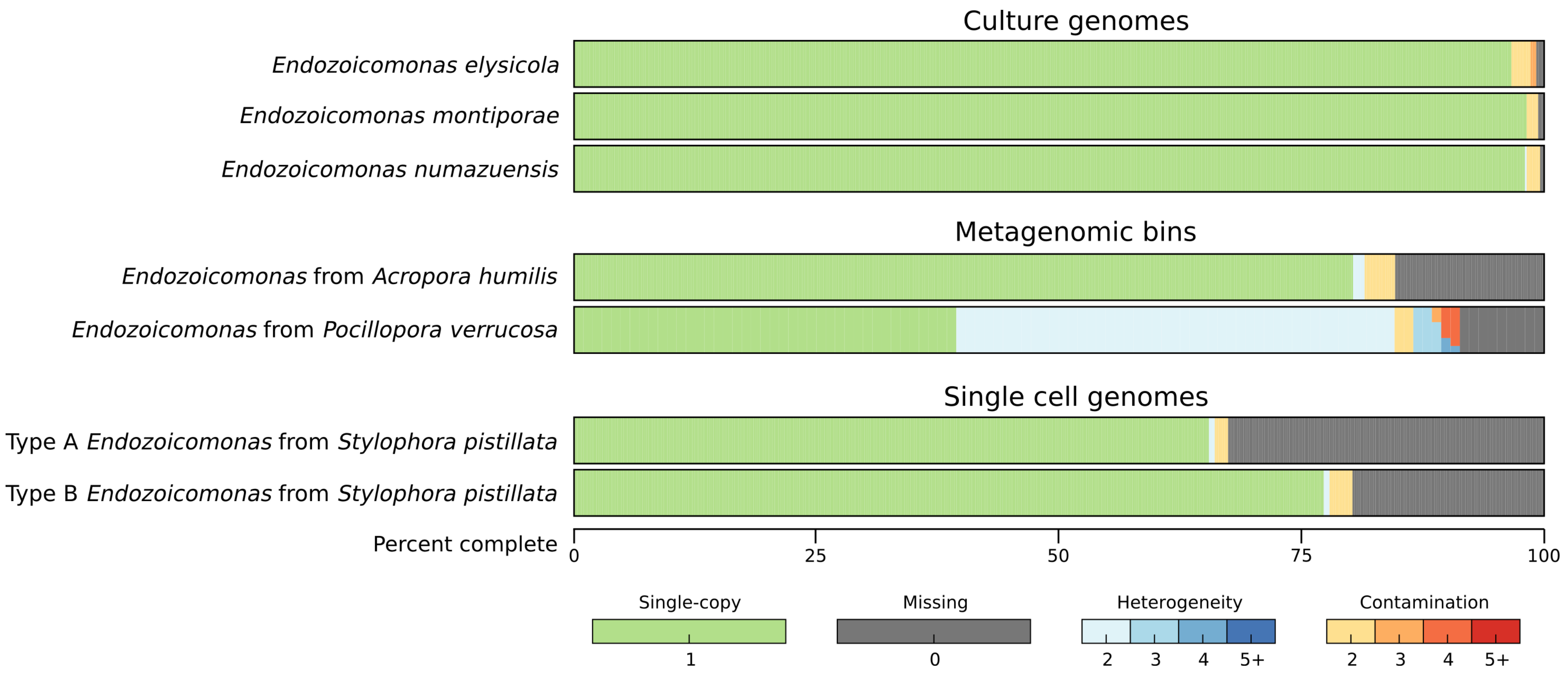

Supplementary Figure 1. Completeness and contamination of the *Endozoicomonas* genomes obtained using cultured isolates, metagenomic binning and single cell genomics. The completeness and contamination estimates are based on essential single copy genes, which are expected to be present in all genomes.

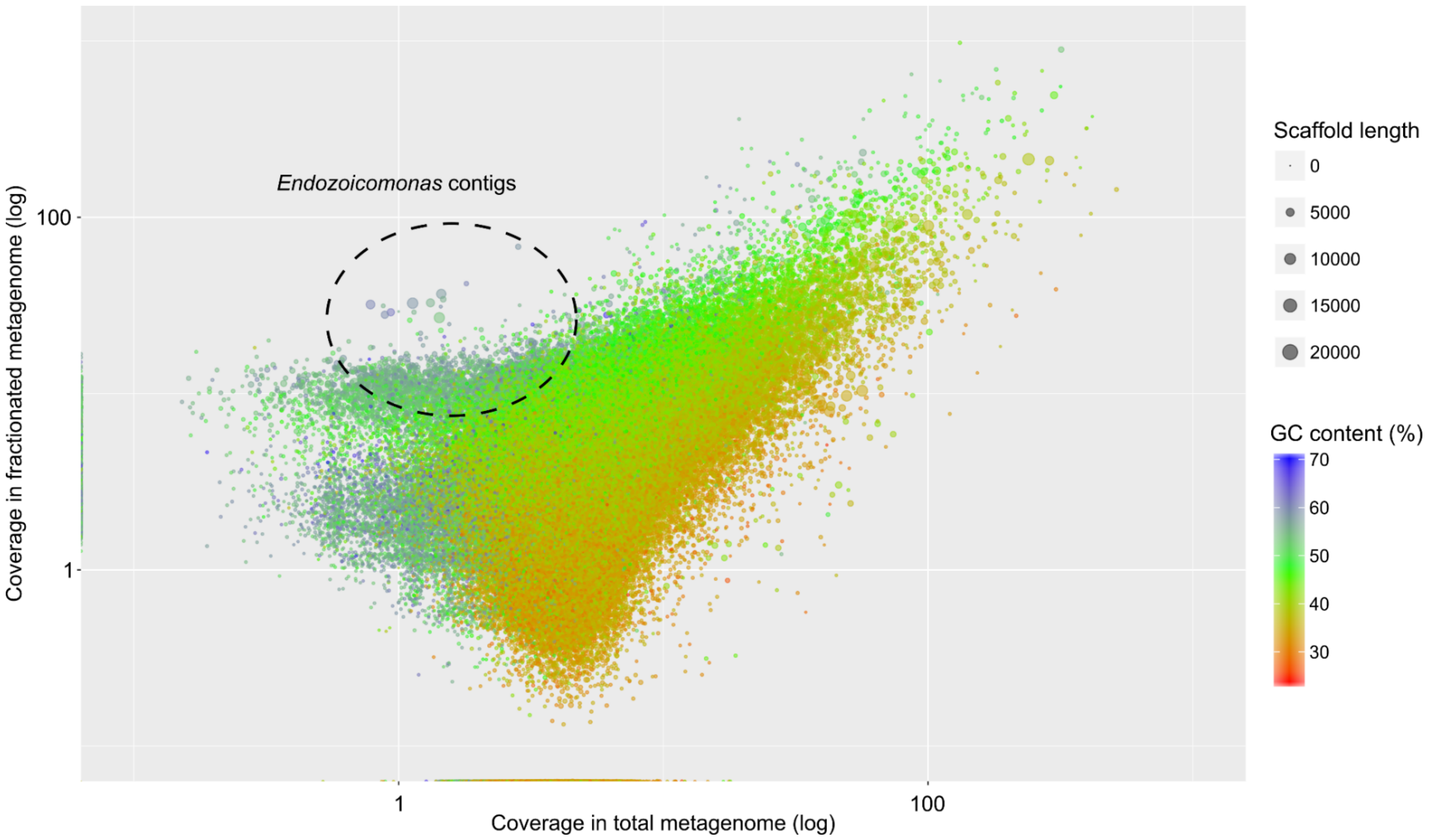

Supplementary Figure 2. Example of binning *Endozoicomonas* contigs from a metagenome of the coral *Pocillopora verrucosa*. *Endozoicomonas* contigs are differentiated by a higher GC content and smaller physical size (i.e. higher coverage in the fractionated metagenome). The location of *Endozoicomonas* contigs is confirmed later in the binning procedure by overlaying essential single copy genes.

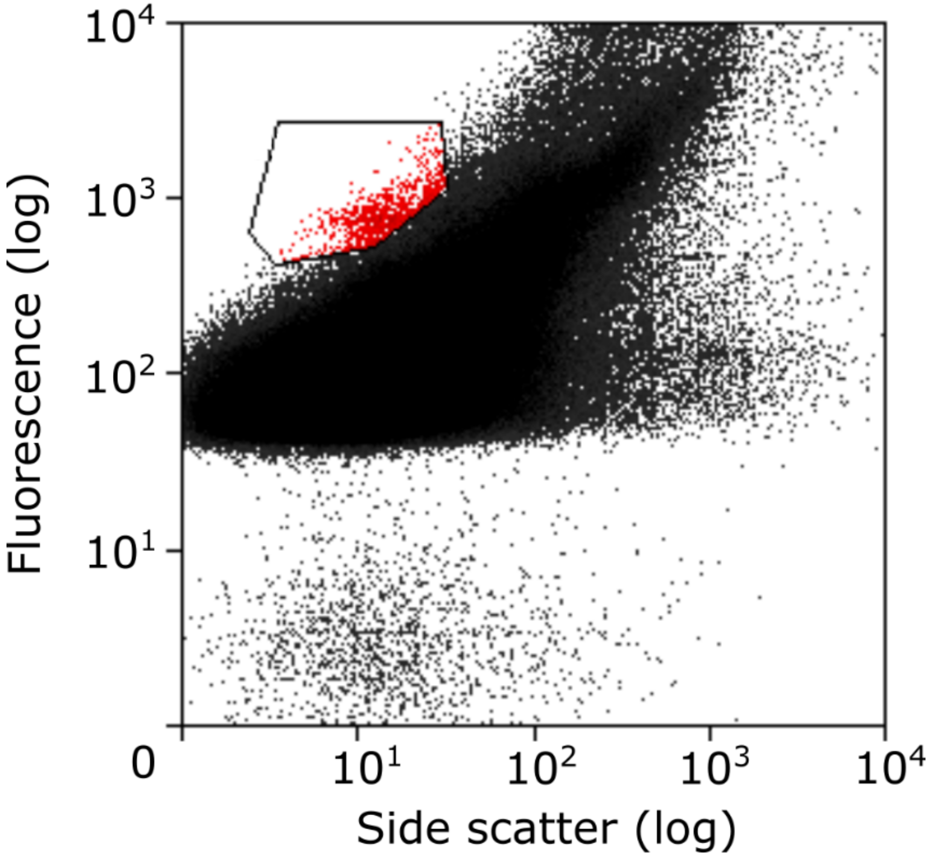

Supplementary Figure 3. Fluorescent activated cell sorting (FACS) of homogenized coral cells. Fluorescence intensity is plotted against side-scatter intensity and cells that are within the expected bacterial size parameters (colored red) were collected for further testing and identification.

Supplementary Table 1. HiSeq sequence statistics including number of final reads used for *Endozoicomonas* genome assemblies.

Genome	Technique	Total reads	Cleaned reads	Final assembled reads (after binning)
Endozoicomonas from *Stylophora pistillata* (Type A; 10 cells)	Single cell genomics	94,915,874	89,901,144	89,901,144
Endozoicomonas from *Stylophora pistillata* (Type B; 3 cells)	Single cell genomics	27,630,137	25,971,750	25,971,750
Endozoicomonas from *Acropora humilis*	Metagenomics	25,640,269	21,531,090	138,898
Endozoicomonas from *Pocillopora verrucosa*	Metagenomics	21,704,023	16,871,594	1,021,516

Supplementary Table 2. Number of genes annotated into each RAST subsystem classification.

Subsystem	*Endozoicomonas elysicola*	*Endozoicomonas* from *Acropora humilis*	*Endozoicomonas montiporae*	*Endozoicomonas numazuensis*	*Endozoicomonas* from *Stylophora pistillata* (Type A)	*Endozoicomonas* from *Stylophora pistillata* (Type B)	*Endozoicomonas* from *Pocillopora verrucosa*
Amino Acids and Derivatives	465	155	359	349	103	174	260
Carbohydrates	439	111	337	362	94	149	255
Cell Division and Cell Cycle	25	21	34	24	46	36	48
Cell Wall and Capsule	168	71	137	170	102	87	97
Cofactors, Vitamins, Prosthetic Groups, Pigments	284	93	279	297	73	146	282
DNA Metabolism	110	59	152	143	105	97	110
Dormancy and Sporulation	3	2	4	3	2	3	6
Fatty Acids, Lipids, and Isoprenoids	155	44	120	155	52	59	114
Iron acquisition and metabolism	33	9	10	32	23	19	7
Membrane Transport	149	79	132	181	46	61	83
Metabolism of Aromatic Compounds	19	13	37	16	4	6	12
Miscellaneous	21	5	23	22	16	14	19
Motility and Chemotaxis	10	0	75	74	4	13	35
Nitrogen Metabolism	31	10	29	33	3	4	8
Nucleosides and Nucleotides	146	56	121	110	27	56	105
Phages, Prophages, Transposable elements, Plasmids	6	1	32	14	5	10	7
Phosphorus Metabolism	44	29	47	50	23	36	50
Photosynthesis	0	0	0	0	0	0	0
Potassium metabolism	26	10	17	19	18	13	21
Protein Metabolism	251	82	263	258	112	196	152
RNA Metabolism	126	66	180	195	80	88	202
Regulation and Cell signaling	65	28	43	75	12	24	52
Respiration	117	49	121	107	63	67	97
Secondary Metabolism	4	4	4	4	0	0	0
Stress Response	148	45	109	138	39	66	118
Sulfur Metabolism	51	7	25	32	10	10	19
Virulence, Disease and Defense	96	30	83	68	25	39	55

Supplementary Table 3. Number of genes in the RAST "amino acid and derivatives" classification.

Subsystem	*Endozoicomonas elysicola*	*Endozoicomonas* from *Acropora humilis*	*Endozoicomonas montiporae*	*Endozoicomonas numazuensis*	*Endozoicomonas* from *Stylophora pistillata* (Type A)	*Endozoicomonas* from *Stylophora pistillata* (Type B)	*Endozoicomonas* from *Pocillopora verrucosa*
Alanine, serine, and glycine	48	16	48	16	21	34	19
Arginine; urea cycle, polyamines	104	3	73	3	40	50	105
Aromatic amino acids and derivatives	56	30	65	30	11	18	51
Branched-chain amino acids	115	53	49	53	0	0	7
Glutamine, glutamate, aspartate, asparagine	28	17	25	17	9	11	16
Histidine Metabolism	15	6	17	6	0	4	0
Lysine, threonine, methionine, and cysteine	80	24	66	24	20	49	51
Proline and 4-hydroxyproline	16	4	14	4	2	8	11
